# The association of cold weather and all-cause and cause-specific mortality in the island of Ireland between 1984 and 2007

**DOI:** 10.1186/1476-069X-13-104

**Published:** 2014-12-06

**Authors:** Ariana Zeka, Stephen Browne, Helen McAvoy, Patrick Goodman

**Affiliations:** Institute for the Environment, Brunel University London, London, UK; Institute of Public Health Ireland, Dublin, Ireland; Environmental Health Sciences Institute, Dublin Institute of Technology, Dublin, Ireland

**Keywords:** Cold weather, Case-crossover, Cardiovascular, Distributed lags, Mortality, Respiratory, Stroke

## Abstract

**Background:**

This study aimed to assess the relationship between cold temperature and daily mortality in the Republic of Ireland (ROI) and Northern Ireland (NI), and to explore any differences in the population responses between the two jurisdictions.

**Methods:**

A time-stratified case-crossover approach was used to examine this relationship in two adult national populations, between 1984 and 2007. Daily mortality risk was examined in association with exposure to daily maximum temperatures on the same day and up to 6 weeks preceding death, during the winter (December-February) and an extended cold period (October-March), using distributed lag models. Model stratification by age and gender assessed for modification of the cold weather-mortality relationship.

**Results:**

In the ROI, the impact of cold weather in winter persisted up to 35 days, with a cumulative mortality increase for all-causes of 6.4% (95% CI = 4.8%-7.9%) in relation to every 1°C drop in daily maximum temperature, similar increases for cardiovascular disease (CVD) and stroke, and twice as much for respiratory causes. In NI, these associations were less pronounced for CVD causes, and overall extended up to 28 days. Effects of cold weather on mortality increased with age in both jurisdictions, and some suggestive gender differences were observed.

**Conclusions:**

The study findings indicated strong cold weather-mortality associations in the island of Ireland; these effects were less persistent, and for CVD mortality, smaller in NI than in the ROI. Together with suggestive differences in associations by age and gender between the two Irish jurisdictions, the findings suggest potential contribution of underlying societal differences, and require further exploration. The evidence provided here will hope to contribute to the current efforts to modify fuel policy and reduce winter mortality in Ireland.

## Background

The impacts of cold weather on human health are increasingly being evidenced by epidemiologic studies, contributing to a wide range of public health outcomes including increased mortality from cardiovascular, cerebrovascular and respiratory diseases [1–3]. Under the influences of global climate change, both mean temperature and temperature variability are expected to increase globally, likely affecting the increasing frequency of weather extremes [4, 5]. Recent record low temperatures in Northern Europe and the United States highlight the potential health and societal impacts of extreme winter weather, despite the rise in global average temperatures [3, 6]. The large death toll and the disruption associated with the cold weather in Europe in the winter of 2005–2006 was a timely reminder of how poorly prepared many populations were to the dangers of extreme cold temperatures [7].

Although there has been a general trend of rising average temperatures in Ireland over the past 30 years, the island has witnessed extreme cold weather events over the same period [8, 9]. Taking into consideration the differences in underlying population health, public policies targeted at preventing winter mortality, health care provision, and socioeconomic and demographic construct of the two Irish jurisdictions [10–12], the health impacts of cold weather could potentially differ.

It is therefore important to understand the magnitude and geographic differences of the cold weather-health relationship on the island of Ireland in order to inform public policies aimed at improvement of population health, and targeting of vulnerable groups during periods of cold weather. This study aimed to assess the cold temperature and all-cause and cause-specific mortality relationship in the Republic of Ireland (ROI) and Northern Ireland (NI), and to assess for modification of this relationship by age and gender.

## Methods

### Mortality

Individual daily deaths for ages ≥18 years were obtained from the Irish Central Statistics Office for data in the ROI, and Northern Ireland Social Research Agency for data in NI for the period of January 1^st^ 1984 and December 31^st^ 2007. International Classification of Disease-9 (ICD-9) codes were used up to 2006, and ICD-10 codes from 2007. Non-accidental deaths were used for this study (ICD-9 codes 001-799/ICD-10 codes A00-R99), further categorised to primary cause-specific mortality for cardiovascular disease (CVD) (390–429; I01-I52), ischaemic heart disease (IHD) (410–414, 429.2; I20-I52), myocardial infarction (MI) (410; I21-I22), respiratory disease (460–519; J0-J99), pneumonia (480–486; J12-J18), chronic obstructive pulmonary disease (COPD) (490–492, 494–496; J40-J44, J47), and stroke (430–438; I60-I69).

### Weather data

Full time-series weather data for the study period were obtained from Met Eireann, for ten weather stations in the ROI; and from the United Kingdom Meteorological Office for six weather stations in NI. The data included daily maximum, minimum, and mean temperatures, relative humidity and atmospheric pressure, and were merged on daily mortality data by county, based on geographical proximity to weather stations.

### Statistical analyses

A time-stratified case-crossover approach was applied to assess the associations between cold weather and daily mortality in each of the two Irish jurisdictions. The case-crossover design has been originally developed and used in air pollution studies, and has been extensively described elsewhere [13–15]. Briefly, the approach is a variant of the case–control study, comparing each person’s exposure at the time of death (case time) with that person’s exposure in other times (control times) [13]. A time-stratified approach is used to select control days, with the day of death chosen as the case day, and controls chosen as all other days, the same day of the week, in the same month and year as the case day, leaving two days between each control to eliminate any serial correlation [14, 15]. An advantage of this design is that, if control days are chosen close in time to the day of death, there is no confounding by slowly varying personal characteristics, and even very strong confounding by season and time trend can be removed [16–18]. Conditional logistic regression was applied to matched pairs to compare the different characteristics between the case day and its control days [13]. The choice of the modeling approach was determined after data were tested that model assumptions were met, and no important data overdispersion was present [19].

### Definition of seasons

Data were analyzed for the winter season (December-February) and an extended cold period (October-March). To investigate whether the observed cold weather-mortality effects were not just confined to winter months, separate analyses were also carried out for the other months in the extended cold period (October, November, and March). Cold weather-related mortality increases were observed during the extended cold period; however these associations were weaker and less persistent than those observed in winter, and will not be discussed further.

### Temperature metrics

Different temperature definitions have been used in literature with no uniform criteria used to identify the best cold weather exposure metric [20, 21]. In this study, daily maximum, mean and minimum temperatures were tested as exposure metrics in the models. Strongest mortality associations were obtained with daily maximum temperature, with only small differences between this and associations with mean temperature; these were slightly greater than those observed for minimum temperature. It was, therefore, decided to present results only for maximum temperature. To assess the impact of temperature variability on mortality, associations with temperature differences between daily maximum and minimum temperature, daily maximum and mean temperature, and weekly mean maximum and minimum temperature were examined as independent variables in the models. None of these associations were statistically important, and thus are not presented here.

### Definition of exposure variables

Daily mortality in association with exposure to temperatures on the same day of the death (lag0) up to 6 weeks prior to the death (lag1-42 days) was examined. Weekly means of daily maximum temperatures were calculated for each of the 6 weeks, for lag1-7, 8–14, 15–21, 22–28, 29–35, and 36–42 days. Distributed lag models included all weekly lags, with lag0 included as an independent variable. This model is a way to estimate unbiased time trends of the exposure-response relation, by controlling for confounding in temperature-mortality associations in one lag structure by exposure to cold temperature in other lags [17]. Models were also tested with a finer definition of lag structure (means of lag1-2, 3–5, 6–9, 10–14, 15–21, 22–28 and 29–35 days) to examine acute, medium and longer term effects of temperatures. However, the models were more efficient with fewer lag structures and overall results were similar. All models included ‘day of the week’ as an indicator variable, with Monday as the reference, relative humidity and atmospheric pressure averaged over 3 days: the day of death and two days prior. No important contributions of relative humidity and atmospheric pressure were found in other lag structures.

No associations were observed between any mortality cause and temperature in week 6 prior to death (lag35-42 days) in both jurisdictions. Important associations were observed with temperatures up to 35 days before death for the ROI, and up to 28 days for NI during winter, and only those were presented here.

### Cumulative mortality increase estimate calculation

Estimates for important associations between daily mortality and lags of weekly average of maximum temperature prior to death were used to calculate cumulative estimates of mortality increases by summing the coefficients. Cumulative estimates did not include the association of mortality with temperature on the same day (lag0). This association is consistent with previous studies that assessed the cold weather-mortality relationship [1, 22], where lag0 was positive and potentially suggestive of warm temperature effects. The overall cumulative estimate variance was computed using previously described algorithms [23].

### Modifying variables

The modification of the temperature-mortality relationship was examined by stratifying the models by gender, and age in the following groups: ≥18-64, ≥65-74, and ≥75 years. Statistically important differences between cumulative estimates of strata of a potential modifier were tested by calculating the 95% confidence interval of this difference (95% CI) [24]. It is also possible that the response of the two populations to the cold temperatures has changed over time. To understand long term patterns of the cold weather-mortality association, additional analyses were carried out stratified for different time periods. In these analyses, some differences over time, with effects slightly diminishing in more recent years, were observed for both jurisdictions (data not shown).

The study did not consider adjustment for air pollution; these data were also inconsistent across the Island for the study period. Current practice in epidemiologic studies examining the weather and health association is to automatically adjust for air pollution, if information on this is available. In a recent article, Buckley et al. indicated that there is no known mechanism by which air pollution confounds the cold temperature and mortality (health) relationship [25]. In support of this rationale, previous epidemiologic studies show little or no influence of air pollution on the cold weather-mortality relationship [1–3, 22, 26–28]. This evidence emphasizes the robustness of our study findings, which are very important from a public policy perspective.

Results are presented as percentage (%) change in mortality per 1°C decrease in weekly average of daily maximum temperature.

## Results

From the 24 years of the study data, there were a total of 709,110 non-accidental deaths in the ROI, and 347,936 in NI (Table 1). Of these deaths in the ROI, 33% were from CVD causes, 15% from respiratory diseases, and 9% from stroke, with NI showing a similar distribution. In both jurisdictions, more males died amongst ages ≥18 and 74 years; female deaths were greater in the ≥75 years age group. More than half of the deaths occurred in the ≥75 years old.

**Table 1:** **Total number of deaths stratified by age and gender in the Republic of Ireland and Northern Ireland between 1984 and 2007**

Cause of death	Gender	Republic of Ireland								Northern Ireland							
		All year				Cold period				All year				Cold period			
		All ages	18-64 yr.	65-74 yr.	75+ yr.	All ages	18-64 yr.	65-74 yr.	75+ yr.	All ages	18-64 yr.	65-74 yr.	75+ yr.	All ages	18-64 yr.	65-74 yr.	75+ yr.
All-cause Mortality	Male	367548	82345	100866	184337	196918	39411	53776	93136	168304	40129	46253	81922	89471	19248	24425	40922
	Female	341562	52572	65219	223771	184181	24471	34704	116771	179632	26934	33587	11911	96612	12723	17801	62179
CVD	Male	127771	28183	37217	62371	68745	14677	20026	31487	57434	13868	17520	26046	30595	7168	9328	12939
	Female	103469	8220	18991	76258	56196	4312	10210	39949	51892	4897	10566	36429	28140	2684	5738	18798
IHD	Male	101936	23909	31549	46472	54697	12450	16910	23309	44975	11844	14243	18888	23990	6144	7608	9328
	Female	72103	5997	14898	51206	39066	3167	7989	26636	34911	3581	7889	23441	18932	1980	4243	12061
MI	Male	71695	16429	23351	31912	38526	8551	12516	16001	37543	9046	12208	16289	20063	4693	6515	8058
	Female	49351	4235	11189	33925	26868	2246	6058	17638	30452	2963	7129	20360	16602	1641	3853	10533
Respiratory Disease	Male	54019	5348	12151	36520	31263	2858	7169	19948	26398	3058	5634	17706	15217	1550	3264	9635
	Female	52180	3601	7966	40613	30680	2024	4736	22933	31651	2357	4123	25171	18605	1193	2418	14405
COPD	Male	24523	2228	6913	15371	14435	1343	4144	8360	7765	920	2357	4488	4465	568	1358	2344
	Female	16115	1422	4140	10546	9729	889	2570	5892	5627	736	1628	3263	3379	458	996	1794
Pneumonia	Male	21613	1556	3443	16470	12405	868	2018	9042	14113	1081	2193	10839	8163	564	1286	6010
	Female	27636	1029	2559	23933	16051	614	1458	13580	21843	819	1758	19266	12818	416	1015	11115
Stroke	Male	28235	3637	6932	17666	15273	1915	3719	9033	14781	1951	3552	9278	7865	1005	1866	4662
	Female	38902	2978	5834	30090	21183	1520	3139	15877	24760	1696	3468	19596	13332	866	1838	10278

Abbreviations: COPD = chronic obstructive pulmonary disease, CVD = cardiovascular disease, IHD = ischemic heart disease, MI = myocardial infarction, total = male and female, and yr. = years.

Overall, on average, temperatures in NI in winter were 1°C less than in the ROI, a trend which was reflected across all temperature metrics (Table 2).

**Table 2:** **Weather descriptives for the Republic of Ireland and Northern Ireland between 1984 and 2007**

		Republic of Ireland					Northern Ireland				
Season	Temperature*	Mean ± SD	Percentiles				Mean ± SD	Percentiles			
			99th	90 ^th^	10 ^th^	1 ^st^		99th	90 ^th^	10 ^th^	1 ^st^
All year	Mean	9.7 ± 4.4	19.0	15.4	4.1	0.4	9.1 ± 4.4	18.6	15.2	3.4	0.0
	Minimum	6.3 ± 4.4	15.2	12.1	0.6	−3.5	5.9 ± 4.5	15.0	11.9	0.0	−3.9
	Maximum	13.0 ± 4.8	24.1	19.4	7.0	3.1	12.4 ± 4.9	23.5	18.9	6.1	2.5
Summer§	Mean	14.7 ± 2.3	20.5	17.8	11.9	9.4	14.4 ± 2.4	20.1	17.5	11.4	8.9
	Minimum	10.9 ± 2.6	16.5	14.0	7.6	4.3	10.6 ± 2.7	16.2	13.9	7.1	3.6
	Maximum	18.6 ± 2.8	26.4	22.3	15.2	12.6	18.1 ± 2.8	25.7	21.9	14.7	12.0
Winter Months^†^	Mean	5.8 ± 2.9	11.8	9.5	2.0	−1.1	4.9 ± 2.7	10.7	8.3	1.5	−1.4
	Minimum	3.1 ± 3.3	10.3	7.4	−1.2	−4.9	2.2 ± 3.2	9.5	6.4	−1.8	−5.6
	Maximum	8.6 ± 2.8	13.9	12.0	4.8	1.5	7.6 ± 2.7	13.2	11.1	4.0	1.0
Cold Period^‡^	Mean	7.0 ± 3.2	14.4	11.1	2.9	−0.4	6.3 ± 3.2	14.0	10.5	2.3	−0.8
	Minimum	4.1 ± 3.7	16.0	8.8	−0.5	−4.4	3.4 ± 3.6	12.0	8.3	−1.0	−4.9
	Maximum	10.0 ± 3.2	17.4	14.0	5.7	2.2	9.1 ± 3.3	16.7	13.2	4.9	1.7

Abbreviations: SD = standard deviation, *in degrees Celsius (°C), ^§^Summer = June to August, ^†^Winter Months = December to February, and ^**‡**^Cold Period = October to March.

Mortality-temperature associations for the winter season in the ROI are presented in Table 3. All-cause mortality showed the greatest increase associated with temperatures in the preceding week to death; the impact of cold temperature on mortality slightly weakened, but lasted up to 5 weeks prior to death. Similar associations were observed for CVD mortality; however, effects were seen up to the preceding 4 weeks. Respiratory mortality increases were strongest in relation to temperatures 2 weeks prior to death, and remained strong in weeks 3 to 4, extending, but slightly weakening into week 5. COPD and pneumonia mortality showed a similar pattern. Stroke mortality increases were observed in association with temperatures up to 3 weeks preceding death.

**Table 3:** Estimated mortality percentage change per 1°C decrease in maximum temperature in the Republic of Ireland during winter^‡^ for ages ≥18 years, 1984–2007*

	Lag of Week 1	Lag of Week 2	Lag of Week 3	Lag of Week 4	Lag of Week 5	Cumulative
	% Change (95% CI)	% Change (95% CI)	% Change (95% CI)	% Change (95% CI)	% Change (95% CI)	% Change (95% CI)
All-cause Mortality	1.8 (1.5, 2.1)	1.4 (1.1, 1.7)	1.4 (1.1, 1.7)	1.0 (0.7, 1.3)	0.8 (0.5, 1.0)	6.4 (4.8, 7.9)^§^
CVD	1.8 (1.3, 2.3)	1.3 (0.8, 1.8)	1.3 (0.8; 1.8)	1.2 (0.6, 1.7)	0.4 (−0.1, 0.9)	5.6 (3.5, 7.7)^∂^
IHD	2.1 (1.5, 2.7)	0.8 (0.2, 1.4)	1.4 (0.8, 2.0)	1.0 (0.4, 1.6)	0.5 (−0.1, 1.1)	5.3 (2.9, 7.8)^∂^
MI	1.9 (1.2, 2.7)	0.8 (0.1, 1.6)	1.4 (0.7, 2.1)	0.9 (0.2, 1.6)	0.7 (−0.03, 1.3)	5.1 (2.2, 8.0)^∂^
Respiratory Disease	2.5 (1.7, 3.2)	3.2 (2.5, 3.9)	2.6 (1.9, 3.4)	2.7 (1.9, 3.4)	1.5 (0.8, 2.2)	12.5 (8.9, 16.2)^§^
COPD	2.3 (1.2, 3.5)	2.8 (1.6, 3.9)	2.3 (1.2, 3.5)	2.9 (1.7, 4.0)	1.4 (0.3, 2.5)	11.7 (6.0, 17.4)^§^
Pneumonia	2.9 (1.8, 4.0)	3.6 (2.6, 4.7)	3.2 (2.2, 4.3)	2.5 (1.5, 3.6)	1.5 (0.5, 2.5)	13.8 (8.4, 19.1)^§^
Stroke	2.5 (1.5, 3.5)	1.1 (0.1, 2.1)	1.4 (0.4, 2.4)	0.8 (−0.2, 1.7)	0.8 (−0.1, 1.8)	5.0 (2.1, 8.0)^†^

Abbreviations: CI = confidence intervals, COPD = chronic obstructive pulmonary disease, CVD = cardiovascular disease, IHD = ischemic heart disease, MI = myocardial infarction. *Models included mortality data for ages ≥18 years, and were adjusted for Lag0 of maximum temperature, relative humidity and atmospheric pressure averaged over 3 days lag, and day of week. % Change = estimated mortality percentage change per 1°C decrease in weekly mean of maximum temperature. Week 1 corresponds to the mean of daily maximum temperature for lag1-7, week 2 for lag8-14, week 3 for lag15-21, week 4 for lag22-28, and week 5 for lag29-35. ^‡^Winter Months = December to February. ^§^Cumulative change calculated for lag of week 1 to 5. ^∂^Cumulative change calculated for lag of week 1 to 4. ^†^Cumulative change calculated for lag of week 1 to 3.

In the ROI, cumulative increases in mortality were calculated over the 5 week period for all-cause and respiratory disease; for a 4-week period for CVD mortality; and over the preceding 3 weeks for stroke. The cumulative increase for all-cause mortality was 6.4% (95% confidence interval-CI = 4.8%-7.9%). Similar cumulative increases were observed for CVD, and stroke mortality; increases for respiratory mortality were twice as large.

Table 4 presents the temperature-mortality associations during the winter months in NI. All-cause mortality showed important increases up to 3 weeks prior to death. The increases for CVD and stroke mortality were strongest in weeks 1 and 3. Respiratory mortality increased for cold temperatures 1 to 4 weeks prior, with similar associations observed for pneumonia. COPD mortality was associated with cold temperatures up to 3 weeks prior.

**Table 4:** Estimated mortality percentage change per 1°C decrease in maximum temperature in Northern Ireland during winter^‡^ for ages ≥18 years, 1984–2007*

	Lag of Week 1	Lag of Week 2	Lag of Week 3	Lag of Week 4	Cumulative
	% Change (95% CI)	% Change (95% CI)	% Change (95% CI)	% Change (95% CI)	% Change (95% CI)
All-cause Mortality	1.7 (1.3, 2.2)	1.2 (0.8, 1.7)	1.6 (1.1, 2.0)	0.4 (−0.01, 0.9)	4.5 (3.2, 5.9)^†^
CVD	1.9 (1.1, 2.7)	0.5 (−0.3, 1.3)	1.5 (0.7, 2.3)	0.1 (−0.7, 0.9)	3.9 (1.5, 6.3)^†^
IHD	2.3 (1.3, 3.2)	0.1 (−0.8, 1.0)	1.5 (0.5, 2.4)	−0.3 (−1.2, 0.6)	3.8 (1.0, 6.6)^†^
MI	2.2 (1.2, 3.2)	−0.02 (−1.0, 1.0)	1.5 (0.5, 2.5)	−0.2 (−1.2, 0.8)	3.7 (0.7, 6.7)^†^
Respiratory Disease	2.8 (1.8, 3.9)	2.7 (1.7, 3.7)	4.3 (3.2, 5.3)	1.4 (0.4, 2.4)	11.2 (7.1, 15.3)^∂^
COPD	3.7 (1.5, 5.8)	3.1 (1.0, 5.2)	3.9 (1.8, 6.0)	0.8 (−1.3, 2.9)	10.6 (4.2, 17.0)^†^
Pneumonia	2.1 (0.8, 3.4)	2.5 (1.2, 3.7)	4.1 (2.8, 5.4)	1.5 (0.2, 2.8)	10.2 (5.0, 15.4)^∂^
Stroke	2.0 (0.7, 3.3)	0.9 (−0.4, 2.2)	1.9 (0.6, 3.2)	0.9 (−0.4, 2.2)	4.8 (0.9, 8.8)^†^

Abbreviations: CI = confidence intervals, COPD = chronic obstructive pulmonary disease, CVD = cardiovascular disease, IHD = ischemic heart disease, MI = myocardial infarction. *Models included mortality data for ages ≥18 years, and were adjusted for Lag0 of maximum temperature, relative humidity and atmospheric pressure averaged over 3 days lag, and day of week. % Change = estimated mortality percentage change per 1°C decrease in weekly mean of maximum temperature. Week 1 corresponds to the mean of daily maximum temperature for lag1-7, week 2 for lag8-14, week 3 for lag15-21, and week 4 for lag22-28. ^‡^Winter Months = December to February. ^∂^Cumulative change calculated for lag of week 1 to 4. ^†^Cumulative change calculated for lag of week 1 to 3.

Cumulative mortality increases were calculated for associations with cold temperatures in the 4 weeks before death for all respiratory disease and pneumonia. For all other causes important effects were only seen up to the preceding week 3 and cumulative increases were only calculated over this period. Cumulative mortality increase for all-cause was 4.5% (95% CI = 3.2%-5.9%), for CVD 3.9% (95% CI = 1.5%-6.3%), respiratory disease 11.2% (95% CI = 7.1%-15.3%), and stroke 4.8% (95% CI = 0.9%-8.8%).

Some differences by age and gender in the cold weather-mortality associations were observed in both jurisdictions; however, overall these differences were not statistically important. Mortality increases in the ROI (Table 5) were mainly observed amongst ≥65 years old, and increased with age for all-cause and CVD. Stroke mortality increases were mainly amongst ≥75 years old, and females. No gender differences were observed for other mortality causes. In NI (Table 6), most of the cold weather-mortality impacts on all-cause and CVD were observed amongst ages ≥65 years; CVD and stroke mortality increases were only observed amongst males.

**Table 5:** Estimated cumulative mortality percentage change per 1°C decrease in maximum temperature by age and gender groups, in the Republic of Ireland during winter^‡^ for ages ≥18 years, 1984–2007*

	Male and Female			All Ages	
	18-64 years	65-74 years	75+ years	Male	Female
	% Change (95% CI)	% Change (95% CI)	% Change (95% CI)	% Change (95% CI)	% Change (95% CI)
All-cause Mortality^§^	3.3 (−0.3, 7.0)	5.2 (2.1, 8.3)	7.7 (5.7, 9.7)	6.1 (4.0, 8.2)	6.6 (4.4, 8.8)
CVD^∂^	3.9 (−1.5, 9.3)	4.8 (0.4, 9.1)	6.3 (3.5, 9.2)	5.6 (2.7, 8.5)	5.5 (2.4, 8.7)
IHD^∂^	3.3 (−2.6, 9.3)	4.7 (−0.1, 9.5)	6.4 (3.0, 9.7)	5.3 (2.1, 8.5)	5.4 (1.6, 9.2)
MI^∂^	4.0 (−3.1, 11.1)	4.0 (−1.5, 9.6)	6.1 (2.0, 10.2)	4.9 (1.1, 8.8)	5.3 (0.8, 9.9)
Respiratory Disease^§^	14.4 (1.8, 27.1)	10.8 (2.8, 18.8)	12.9 (8.6, 17.1)	11.6 (6.5, 16.6)	13.6 (8.5, 18.7)
COPD^§^	15.0 (−3.6, 33.6)	10.5 (0.04, 20.9)	11.8 (4.5, 19.1)	10.2 (2.9, 17.5)	14.0 (5.0, 23.0)
Pneumonia^§^	14.0 (−8.8, 36.6)	9.4 (−5.6, 24.4)	14.4 (8.5, 20.3)	14.2 (6.1, 22.2)	13.5 (6.3, 20.6)
Stroke^†^	2.6 (−7.3, 12.5)	4.2 (−2.9, 11.3)	5.6 (1.7, 9.4)	3.7 (−1.0, 8.4)	5.9 (2.0, 9.9)

Abbreviations: CI = confidence intervals, COPD = chronic obstructive pulmonary disease, CVD = cardiovascular disease, IHD = ischemic heart disease, MI = myocardial infarction. *Models included mortality data for ages ≥18 years, and were adjusted for Lag0 of maximum temperature, relative humidity and atmospheric pressure averaged over 3 days lag, and day of week. % Change = estimated mortality percentage change per 1°C decrease in weekly mean of maximum temperature. ^‡^Winter Months = December to February. ^§^Effect modification examined for cumulative change for lags of week 1 to 5; (∂) for cumulative change for lags of week 1 to 4; and (†) for cumulative change for lags of week 1 to 3.

**Table 6:** Estimated cumulative mortality percentage change per 1°C decrease in maximum temperature by age and gender groups, in Northern Ireland during winter^‡^ for ages ≥18 years, 1984–2007*

	Male and Female			All Ages	
	18-64 years	65-74 years	75+ years	Male	Female
	% Change (95% CI)	% Change (95% CI)	% Change (95% CI)	% Change (95% CI)	% Change (95% CI)
All-cause Mortality^†^	2.7 (−0.7, 6.0)	4.5 (1.6, 7.3)	5.1 (3.3, 6.9)	4.8 (2.8, 6.8)	4.3 (2.4, 6.2)
CVD^†^	4.3 (−1.7, 10.3)	4.7 (−0.1, 9.6)	3.5 (0.1, 6.8)	5.1 (1.8, 8.5)	2.6 (−0.9, 6.1)
IHD^†^	3.6 (−3.0, 10.3)	5.2 (−0.3, 10.6)	3.1 (−0.9, 7.2)	4.4 (0.6, 8.2)	3.1 (−1.3, 7.4)
MI^†^	4.0 (−3.5, 11.6)	4.3 (−1.6, 10.3)	3.3 (−1.2, 7.8)	4.1 (0.02, 8.3)	3.2 (−1.3, 7.8)
Respiratory Disease^∂^	15.3 (0.5, 30.2)	12.8 (2.3, 23.2)	10.4 (4.9, 15.8)	9.2 (3.0, 15.4)	12.9 (7.2, 18.6)
COPD^†^	11.4 (−7.0, 29.7)	15.6 (2.6, 28.6)	7.8 (−2.0, 17.6)	10.4 (1.7, 19.2)	10.9 (0.7, 21.1)
Pneumonia^∂^	19.2 (−6.3, 44.7)	9.5 (−7.6, 26.6)	9.7 (2.4, 17.1)	7.4 (−1.1, 15.9)	11.9 (5.0, 18.8)
Stroke^†^	6.2 (−8.0, 20.3)	3.3 (−6.8, 13.5)	5.0 (−0.3, 10.4)	6.8 (0.2, 13.4)	3.6 (−1.5, 8.7)

Abbreviations: CI = confidence intervals, COPD = chronic obstructive pulmonary disease, CVD = cardiovascular disease, IHD = ischemic heart disease, MI = myocardial infarction. *Models included mortality data for ages ≥18 years, and were adjusted for Lag0 of maximum temperature, relative humidity and atmospheric pressure averaged over 3 days lag, and day of week. % Change = estimated mortality percentage change per 1°C decrease in weekly mean of maximum temperature. ^‡^Winter Months = December to February. ^∂^Effect modification examined for cumulative change for lags of week 1 to 4; and (†) for cumulative change for lags of week 1 to 3.

## Discussion

This study observed strong associations between exposure to cold weather temperatures and mortality in two adult populations, in two Irish jurisdictions over a period of 24 years. Effects of cold weather were overall less persistent in NI than in the ROI. For most associations similar mortality patterns were observed for both jurisdictions. All-cause mortality associations were strongest in the week before death, weakening but extending up to 3 weeks in NI and 5 weeks in the ROI. A similar pattern was observed for CVD mortality, but effects were smaller and less persistent in NI than in the ROI. Cold weather up to 5 weeks before death in the ROI and up to 4 weeks in NI was associated with increases in all respiratory mortality; these increases were more extended and larger than for any other mortality group and cumulative increases were comparable in both jurisdictions, also consistent with findings of previous studies [1, 2, 22, 29]. Stroke mortality increases were seen for exposures up to 3 weeks prior to death, and associations were comparable in both jurisdictions. Overall the impacts of cold temperature on mortality were considerably higher and more persistent in winter, although some effects were still detectable in the other months of the extended cold period.

Different temperature metrics were examined in this study, and the strongest associations were observed for maximum temperature. These findings are in agreement with recent studies which suggest that maximum temperature is more likely to capture days that are consistently cold and extreme cold days [30, 31].

The findings here are also consistent with a previous Irish study [22] which examined the relationship between cold weather and mortality in Dublin over a period of 17 years, using Poisson regression models. They reported mortality increases for all-cause (2.6%), CVD (2.5%), and respiratory disease (6.7%) in relation to 1°C decrease in mean daily temperature up to 40 days preceding death. Similar results were observed in Scotland for all-cause, CVD and respiratory mortality with cold temperatures during the month prior death [29, 32]. Multi-city studies in Europe and the US reported similar increases for all-cause and cause-specific mortality from cold weather during winter and persistence of these associations, with similar lag periods as presented here [1, 2].

Only a small number of studies have looked at the association between cold temperature and mortality from COPD, pneumonia and stroke. An earlier study of 12 cold-climate US cities did not observe any increase in COPD mortality in relation to cold, but their reported increase for pneumonia mortality is comparable with those reported here [26]. Another US study reported elevated risk of dying of COPD associated with cold weather, in an elderly population [33]. Analitis et al. reported increases in stroke mortality associated with cold temperatures over 15 days before death [1], and other studies in Russia and Asia reported similar findings [31, 34, 35].

Weaker associations for CVD and stroke mortality were observed in relation to cold temperatures in the second week before death, with increases in mortality recovering in the week following. This phenomenon has been described previously as harvesting, which is likely due to the depletion in numbers by premature mortality of those most susceptible to cold weather [22, 36, 37]. The extended cold weather-mortality associations observed in our study suggest that there is potentially a cumulative health effect of cold weather and gradual weakening and displacement by death [22, 36, 37], also evidenced by a small number of epidemiologic studies that have considered long lag structures [1–3, 22, 32, 38].

Some suggestive differences in the cold weather-mortality associations by age and gender were observed in both jurisdictions. Corroborating our study findings, Analitis et al. and Goodman et al. reported increasing cold temperature-mortality associations with age [1, 22]; age or gender differences of this relationship have not been confirmed by other studies [7, 39].

This study observed some small differences in the associations of cold weather and mortality between the two Irish jurisdictions. The fact that the island of Ireland is relatively small, with similar weather, population characteristics and demographics, then any observed differences in the population response to the exposure to cold weather is most likely due to different public policies, socioeconomic construct, and health care systems in the two jurisdictions. Currently, there are winter fuel allowances and cold weather payments which target vulnerable population groups in both jurisdictions; however, these schemes differ substantially from each other [40–44]. The effectiveness of these cold weather and winter fuel payments in NI at reducing winter mortality have not been examined; in the ROI it has been suggested that the fuel allowance was not sufficient in meeting home-heating costs, and this was likely due to the contribution of poor thermal efficiency and low household income [11]. Rising fuel prices and demographic changes, combined with the economic recession have contributed to high levels of fuel poverty in the ROI, especially in relation to vulnerable households such as older people, those living alone and lone parent households [10, 12, 45, 46]. The jurisdictional differences in housing stock, insulation and heating, also related to socioeconomic deprivation, influence the extent of fuel poverty [11, 46, 47]. In addition, differences in the identification of the ‘fuel poor’ and ‘those most in need’ can also impact upon the effectiveness and differences of the schemes in the two jurisdictions [46, 48, 49].

A very strong relationship has been observed between the incidence of fuel poverty, social class, geographic and demographic patterns of those most susceptible on the island of Ireland [11, 47]. In both jurisdictions, the number of older people vulnerable to ill-health from cold homes will increase as part of significantly aging population [10]. Currently, there is a concentration of fuel poverty amongst rural older persons households in NI; fuel poverty is highest in most urbanized and very rural areas in the ROI [10, 11]. A greater proportion of older people in NI live alone and in social housing when compared to the ROI [10]. Jurisdictional differences in general population health status, provision of and access to health care, and distribution of health and social inequalities, potentially contribute in concurrence with these policies, and may explain some of the observed differences in the cold weather-mortality associations.

It is important to recognize that climate change induced weather patterns will affect the long term cold weather-mortality relationship. Research suggests that increasing global mean temperatures will do little to reduce morbidity and mortality in winter [4, 6, 50, 51], likely due to increases in temperature variability and weather extremes. These weather patterns have also been observed on the island of Ireland in the past few decades [8, 9]; on this basis only, it may be possible that winter mortality will change over time. However, whether this change will be an increase or decrease is much more complex and multifaceted, and will depend on how rapidly the climate changes, how quickly the population adapt, and on infrastructural and policy interventions, and remains a challenge particularly in view of the aging population on the Island. To understand long term patterns of the cold weather-mortality association, additional analyses were carried out to examine this relationship for different time periods in each jurisdiction. Findings suggested a slight diminishing of the cold weather-mortality relationship in both jurisdictions over time. These findings however, need to be interpreted with caution, considering the complexity of the relationship of mortality and cold winters and the influence of the societal factors discussed earlier.

## Conclusions

This study found strong associations between cold temperatures and mortality with impacts of cold weather persisting up to several weeks, in both Irish jurisdictions. The findings show that increased mortality from cold weather on the island of Ireland is an important and topical public health issue. As the island of Ireland currently has the highest levels of excess winter mortality in Europe, with an estimated 2,800 excess deaths during each winter [12], the key challenges are to develop and implement policies which tackle fuel poverty and reduce winter morbidity and mortality. The fact that some differences in cold weather-mortality patterns are observed between the two jurisdictions, albeit small, suggests that policies and their implementation, and other societal factors potentially play a role in determining population health patterns.

## Abbreviations

°C: Degrees celsius
CI: Confidence intervals
COPD: Chronic obstructive pulmonary disease
CVD: Cardiovascular disease
ICD: International classification of diseases
IHD: Ischemic heart disease
MI: Myocardial infarction
NI: Northern Ireland
ROI: The Republic of Ireland
SD: Standard deviation.
